# Black girls and referrals: racial and gender disparities in self-reported referral to substance use disorder assessment among justice-involved children

**DOI:** 10.1186/s13011-022-00462-6

**Published:** 2022-10-14

**Authors:** Micah E. Johnson, Shawnta L. Lloyd, Skye C. Bristol, Amy L. Elliott, Linda B. Cottler

**Affiliations:** 1 Department of Mental Health Law and Policy, College of Behavioral and Community Sciences, University of South Florida, Tampa, FL USA; 2 Department of Epidemiology, College of Public Health and Health Professions and, College of Medicine, University of Florida, PO Box 100231, Gainesville, FL 32610 USA; 3 Sure Med Compliance, 561 Fairhope Ave, Suite 203 C, Fairhope, AL 36532 USA

**Keywords:** Juvenile, Gender health disparities, Substance use disorder services, Intersectionality

## Abstract

**Background:**

There is a higher prevalence of substance use disorder (SUD) among justice-involved children (JIC). It is critical to ensure that JIC who report current use are referred for SUD assessment and potentially life-saving treatment services. Prior research suggests that certain minoritized groups may be less likely to have ever been referred for screening, and research on intersectionality suggests that these disparities may be exacerbated for racially minoritized females.

**Methods:**

Multivariate logistic regression and interaction effects were employed to analyze longitudinal data from the Florida Department of Juvenile Justice on 12,128 JIC who reported SU in the past 6 months. The main and interaction effects of race and gender on the odds of having a history of reporting a referral to SUD assessment were tested. The primary outcome variable was a self-reported measure of a youth’s history of being referred to service. The control variables included substance type, household income, current SU problems, history of mental health problems, number of misdemeanors, risk to recidivate, and age at first offense.

**Results:**

There were no significant differences in the likelihood of having a history of reporting being referred to SUD assessment between White females, White males, and Latinx females. However, Black females (AOR = 0.62), Latinx males (AOR = 0.71), and Black males (AOR = 0.65) were significantly less likely to self-report having a history of being referred than White males. Black females were 34% likely to report a history of being referred as White males and females.

**Conclusion:**

In this sample, Black females who use substances were substantially less likely to self-report being referred to SUD screening. According to officials, FLDJJ has solid process to ensure referrals are made. Therefore, the results are likely to be due to external factors and alternative explanations. Community leaders and stakeholders may consider culturally relevant and gender-sensitive programs to expand access to services for minoritized adolescents in their communities, schools, and other institutions.

## Background

The failure to refer children who report current substance use (SU) to adequate substance use treatment programs is a serious public health concern, as prolonged SU increases the likelihood of substance use disorder (SUD) and other serious health conditions [1]. Undiagnosed SUD leads to untreated SUD, substantially contributing to the current substance use epidemic and substance trafficking crises in the United States (US). Herein, the construct SU refers to non-medical and/or illicit consumption of substances. Adolescent SU and untreated SUD are associated with violence [2], criminality [3, 4], risky sexual behavior [5], mental health problems [6, 7], justice-involvement and recidivism [8, 9], and other adverse outcomes [10].

In the US, it is estimated that out of 1.03 million adolescents between the ages of 12 and 17 diagnosed with SUD, nearly 969,000, or 75% did not receive substance use treatment [11]. This statistic only reports for properly assessed and diagnosed adolescents and does not account for those misdiagnosed or never diagnosed. The comorbidity of mental illness and SUD increases the likelihood of misdiagnosis and ineffective use of resources. Evidence indicates that justice involved children (JIC), in particular, are grossly under-assessed for SUD. Many JIC are never referred for SUD assessment, even when current SU and associated problems are reported [12–15]. Referral for SUD assessment is a critical point at which youth are assessed, diagnosed, and, if needed, provided with treatment and services. Many youth do not have access to services in their community. The justice system is arguably the de facto drug treatment system for adolescent users in the U.S.

For many youths, their time in the justice system may be the only opportunity to be properly assessed, diagnosed, and treated. However, racial disparities in services can still occur at various points in the system [16, 17]. Black and Latinx JIC are especially underserved due to interpersonal and institutional discrimination and other social factors [18–20]. These disparities may be exacerbated for minoritized girls. Research on the interaction effects of race and gender is needed.

Women and girls are often intentionally influenced to consume illicit substances by peers and sexual predators. Justice-involved females have higher risks of being exploited for sex, prostitution, and drug trafficking compared to justice-involved males and youth in the general population [21–23]. Black women in the criminal justice system have a greater risk for childhood and adult sexual trauma as well as substance use [24]. Addressing these public health issues is essential as women and girls often fill childbearing, caregiving, and leadership roles in their families, especially in Black and Latinx families. So, the biosocial consequences of untreated SUD among girls can proliferate to damage the entire family, community, and future generations.

Women and girls are more likely to face barriers to SUD assessment and treatment services than men, including economic barriers, family responsibilities, and heightened stigma [25–27]. Additionally, sources of mandates for treatment differ for men and women, such that men may have more opportunities to receive treatment. Women are more likely to be referred to treatment by social workers, but men are more likely to be referred by family members, employers, and the criminal justice system [28]. These findings corroborate the need for an intersectional approach to investigating disparities in SUD assessment among JIC.

Intersectionality is a scientific perspective that illuminates the unique experiences of individuals belonging to several disadvantaged groups [29, 30]. Conceptually, minoritized females represent the intersections of two marginalized statuses being female and racial minoritized. In other words, the specific risks associated with a minoritized status, and the female gender have synergistic effects. Historically, intersectionality has illuminated the lived experiences of Black women – a group at the intersection of race and gender that has fundamentally different experiences than those of the same race or gender separately. Applying an intersectional lens, we test if minoritized females report different referral histories than other groups.

Alternatively, disparities in self-reported referrals may be explained by sociodemographic, geographic, and other risk factors that attenuate the effect of race and gender on the likelihood of reporting a referral for SUD assessment [17, 31–33]. Youth may be referred for services by their schools, community, family, or the justice system. Many referral sources consider the youth’s risk of delinquency or recidivism. Certain referral sources may prioritize JIC: a) who use certain substances with higher abuse potential, b) who are from low-income households, c) who are younger in age, d) with current SU problems, e) with a history of mental health problems, f) who have multiple arrests, or g) with a higher recidivism risk score. These factors must also be considered to appropriately investigate potential race and sex disparities in the likelihood of self-reporting that they were referred to SUD services [34, 35].

### The current study

Research indicates that Black and Latinx girls who use substances are at higher risk for SUD and are subject to harsher consequences from lacking appropriate services than their White counterparts [36–38]. However, previous data shows racial and gender disparities in referrals for SUD assessment and services [39–42]. Yet still, the intersection of race and gender has not yet been examined. Using data from FLDJJ, this study investigates the effects of the intersection of race and gender on the likelihood of the youth reporting being referred for an SUD assessment. Applying an intersectionality-informed model, we hypothesized that substance users who are Black or Latinx females would be less likely to report being referred for SUD assessment than substance users who are male or White. To our knowledge, this study is the first to examine the interaction effects of gender and race on self-reported referral for SUD assessment among JIC in Florida. This research fills a striking void in the literature and advances efforts to eliminate health disparities among JIC.

## Methods

### Population

**Fig. 1:**
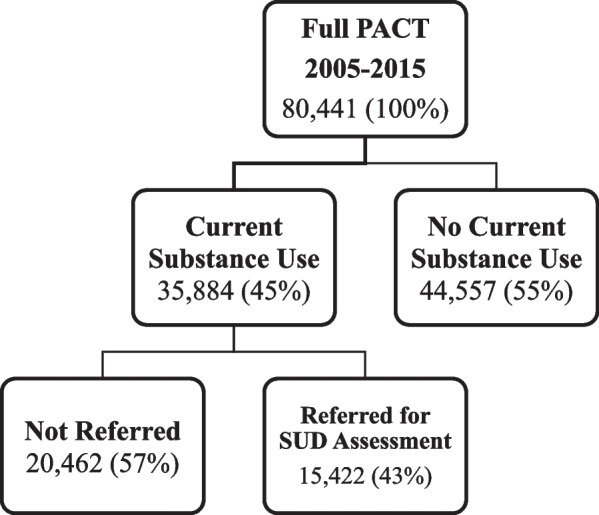
Flow Diagram on History of Self-Reported Referral to SUD Assessment Status. JIA who were not currently using substances were excluded because non-current users do not fit the criteria for referral to SUD assessments. History of Self-Reported Referral to SUD is conditional on current SU. This diagram decribes research methods not FLDJJ processes

The present study leveraged data from FLDJJ, which has collected comprehensive data on all youth entering the FLDJJ system since 2005. The current study includes all youth assessed on three successive occasions with the full assessment. Youth are assessed at intake and every 90 days during supervision. This protocol resulted in three waves: initial intake, 3 months, and 6 months. During intake and in the community, trained FLDJJ staff and counselors administered the Positive Achievement Change Tool (PACT) assessment through in-depth interviews and coded the data using the FLDJJ interface. Those who never reported current SU (neither alcohol nor drugs) were excluded from this sample. A sample of 12,128 JIC met the selection criteria for the study, representing youth who reported using (non-medical or illicit) substances in the past 6 months and had data on each covariate. Substance use ranges from single or experimental use to more frequent and severe use. See the CONSORT diagram in Fig. 1. Among the study sample, 42.5% were White (*n* = 5149), 39.9% of youth were Black (*n* = 4844), 17.6% were Latinx (*n* = 2135), and less than 1.0% were classified as other race (*n* = 161). Approximately 18% of the sample was female.

### Measures

#### History of self-reported referral to SUD assessment

The construct history of self-reported referral to SUD assessment status was operationalized by a dichotomous variable reporting whether or not the youth had ever reported a referral to SUD assessment during or prior to the youth’s involvement with FLDJJ (until May 1st, 2019) measured at the third wave. FLDJJ uses to the Massachusetts Youth Screening Instrument: Second Version (MAYSI-2). Importantly, referrals can also be initiated by the school, community organization, parent, or the youth. These data are collected and maintained by FLDJJ officials. Response values were coded (0) “never referred to SUD assessment” and (1) “yes, referred to SUD assessment.”

#### Race and gender

Race is a construct that commonly includes ethnicity. Race was operationalized via a four-item nominal variable (0 = White, 1 = Black, 2 = Latinx, 3 = other). The nominal variable was converted into dummy variables using the STATA 17 “i.” command to generate three dichotomous variables, Black (0 = White, 1 = Black), Latinx (0 = White, 1 = Latinx), and other (0 = White, 1 = other race) measured at baseline. Gender is a social construct that includes the sex identifications of male and female gender [43]. Gender was self-reported as the “sex of the offender” and categorized as a dichotomous measure (0 = male gender, 1 = female gender) measured at baseline. Race-by-gender was measured via an eight-item nominal variable (0 = White male, 1 = Black male, 2 = Latinx male, 3 = other race male, 4 = White female, 5 = Black female, 6 = Latinx female, and 7 = other race female) that was converted into dummy variables. The” other race” category was excluded from this model due to a small sample size (less than 1%).

#### Covariates

The study controlled for substance type, household income, current SU problems, history of mental health problems, total misdemeanors, recidivism risk level, age at first offense, and history of reporting a referral to SUD assessment at baseline. The construct substance type refers to the types of drugs being currently used. It was measured via a categorical variable recording the types of substances youth reported within the past 6 months. The response options were (0) alcohol only, (1) marijuana only, (2) illicit drugs, and (3) other substance types. The “illicit drugs” category included amphetamines, cocaine/crack, heroin, and other opioids, inhalants, tranquilizers, and hallucinogens. There were limited cases among these options, and therefore they were collapsed into the illicit drug category. The “other substance type” category represented the youth who disclosed a substance type that was not listed on the FLDJJ intake assessment instrument.

Household income was operationalized via a four-item ordinal variable reporting the combined annual income of the youth and their family. Response options were (0) under $15,000, (1) from $15,000 to $34,999, (2) from $35,000 to $49,999, and (3) $50,000 and above. The construct age was operationalized via an ordinal variable reporting the youth’s age at first offense. Response options were (0) Twelve and under, (1) Thirteen to fourteen, (2) Fifteen, (3) Sixteen, and (4) Over sixteen. History of SU problems were measured via a dichotomous variable that reported youths’ current problems related to drug and alcohol use experienced within the past 6 months. The response options were (0) none, meaning no current problems related to drug or alcohol use (including current and past users who have not experienced problems), and (1) yes, current problems related to drug and/or alcohol use. The (1) yes response included those who reported that drug and/or alcohol disrupted school, caused family conflict, etc.

History of mental health problems was measured via a dichotomous variable at intake, reporting the youth’s history of being diagnosed with mental health problems. Response items were (0) ever diagnosed with mental health problems or (1) no history of mental health problems. These data were retrieved from a credentialed authority or self-reported data that was verified by a credentialed authority. Adjudicated misdemeanors were measured via an ordinal variable reporting the number of misdemeanor adjudications in the FLDJJ system. The categories were (0) one or less, (1) two, (2) three or four, and (3) five or more.

The construct recidivism risk level was operationalized via FLDJJ’s overall risk to re-offend measure. FLDJJ uses comprehensive data on criminal history and social factors to calculate a youth’s overall risk to re-offend and corresponding recidivism risk level classification (0 = low, 1 = moderate, 2 = moderate-high, 3 = high). There were no issues with severe collinearity between the recidivism risk level variables and the other covariates in the study (for more information on the FLDJJ risk score, see [44]. The youth’s history of reporting a referral to SUD assessment was controlled at baseline to account for baseline adjustment.

### Analytical procedures

All analyses were completed in STATA 17 SE. Univariate analyses were conducted to describe the data. Bivariate analysis and multivariate logistic regression models were estimated to examine the relationship between race, gender, and control variables and the likelihood of having a history of reporting being referred to SUD assessment. Chi-square tests were used to assess significant associations between all variables and self-reported history of referral. An independent t-test was used to compare the mean age in individuals who had a history of being referred to SUD assessment and those who did not have a history of being referred to SUD assessment. Multivariate models included the covariates described in the “Measures” subsection in Methods (age, household income, substance type used, SU problem, mental health problem, number of misdemeanors, and recidivism risk level). Multiplicative interaction terms were created using the STATA “#” procedure and margins command. The interaction term was interpreted by estimating the predictive margins and plotting the predictive margins to display the relationships graphically. The study uses a complete case analysis; listwise deletion was used to omit cases that did not report current use.

## Results

Table 1 displays the characteristics of JIC stratified by referral status in row percentage. In the sample of JIC who reported current SU, 59% (7110) had a history of reporting a referral to SUD assessment. Among those with a history of a self-reported referral, 27% were White males, 44% were Black males, 13% were Latinx males, 7% were White females, 7% were Black females, and 2% were Latinx females. All variables were significant at the bivariate level, except household income and age at first offense. The majority of JIC who used illicit drugs were polysubstance users. The distribution of substances in the “illicit drugs” category was as follows: amphetamines (.14%), cocaine/crack (.27%), heroin and other opioids (.18%), inhalants (.03%), tranquilizers (.04%) and hallucinogens (.02%), and polyusers (99.32%).

**Table 1:** Characteristics of justice-involved children that reported current substance use who have and have not reported being referred to SUD assessment for substance use

Characteristics	Overall	No history of reporting a referral	History of reporting a referral
	(*n* = 12,128)	(*n* = 5018)	(*n* = 7110)
	n (%)	n (%)	n (%)
	Column percentage	Row percentage	Row percentage
Gender and Race			
White Male	3846 (31.7)	1288 (33.5)	2558 (66.5)
Black Male	4118 (34)	1965 (47.7)	2153 (52.3)
Latinx Male	1803 (14.9)	790 (43.8)	1013 (56.2)
White Female	1303 (10.7)	483 (37.1)	820 (62.9)
Black Female	726 (6)	354 (48.8)	372 (51.2)
Latinx Female	332 (2.7)	138 (41.6)	194 (58.4)
Substance Type(s) Used			
Marijuana Only	7050 (58.1)	3254 (46.2)	3796 (53.8)
Alcohol Only	3093 (25.5)	1245 (40.3)	1848 (59.7)
Illicit Drugs	1131 (9.3)	286 (25.3)	845 (74.7)
Other Drugs	854 (7)	233 (27.3)	621 (72.7)
Household Income			
Under $15,000	3543 (29.2)	1554 (43.9)	1989 (56.1)
$15,000–$34,999	6182 (51)	2601 (42.1)	3581 (57.9)
$35,000–$49,999	1624 (13.4)	591 (36.4)	1033 (63.6)
$50,000 and Over	779 (6.4)	272 (34.9)	507 (65.1)
Past Substance Use Problems			
Yes	4290 (35.4)	1510 (35.2)	2780 (64.8)
Mental Health Problem			
No, History of Problems	9433 (77.8)	3990 (42.3)	5443 (57.7)
Misdemeanors			
1 or Less	6744 (55.6)	3073 (45.6)	3671 (54.4)
2	2908 (24)	1112 (38.2)	1796 (61.8)
3–4	2123 (17.5)	717 (33.8)	1406 (66.2)
5 or More	353 (2.9)	116 (32.9)	237 (67.1)
Recidivism Risk Level			
Low	3892 (32.1)	1907 (49)	1985 (51)
Moderate	2471 (20.4)	987 (39.9)	1484 (60.1)
Moderate-High	3615 (29.8)	1311 (36.3)	2304 (63.7)
High	2150 (17.7)	813 (37.8)	1337 (62.2)
Age at 1st Offense			
12 and Under	2551 (21)	1124 (22.4)	1427 (20.1)
13 to 14	4880 (40.2)	1930 (38.5)	2950 (41.5)
15	2524 (20.8)	986 (19.6)	1538 (21.6)
16	1492 (12.3)	637 (12.7)	855 (12)
Over 16	681 (5.6)	341 (6.8)	340 (4.8)

Model 1 of Table 2 displays the results of multivariate logistic regression models examining the individual main effects of gender and race on the likelihood of self-reporting being referred to SUD assessment while controlling for substance type, household income, past SU problems, history of mental health problems, number of misdemeanors, recidivism risk level and age at first offense. Females (adjusted OR: 0.96, 95% CI: 0.83–1.10), Blacks (adjusted OR: 0.66; 95% CI: 0.58–0.75), and Latinx (adjusted OR: 0.74; 95% CI: 0.64–0.87) were significantly less likely to have reported a referral to SUD assessments than Whites and males, respectively. However, examining the main effects of race-by-gender together revealed more nuanced findings.

**Table 2:** Logistic Regression Estimating Odds Ratios of Referrals by Gender and Race

	Model 1		Model 2	
	Main effects		Gender by race	
	AOR	CI	AOR	CI
*Gender and Race*				
Gender (Ref = Male)				
Female	0.96	[0.83,1.10]		
Race (Ref = White)				
Black	0.66^***^	[0.58,0.75]		
Latinx	0.74^***^	[0.64,0.87]		
Gender by Race (Ref = White Males)				
Black Male			0.65^***^	[0.56,0.75]
Latinx Male			0.71^***^	[0.60,0.85]
White Female			0.91	[0.75,1.09]
Black Female			0.62^***^	[0.49,0.79]
Latinx Female			0.81	[0.59,1.13]
*Covariates*				
Substance Type (Ref = Marijuana Only)				
Alcohol Only	1.11	[0.98,1.27]	1.11	[0.98,1.27]
Illicit Drugs	1.48^***^	[1.20,1.83]	1.48^***^	[1.20,1.83]
Other Drugs	1.48^***^	[1.18,1.86]	1.48^***^	[1.18,1.87]
Income (Ref = Under $15 k)				
$15,000–$34,999	1.06	[0.94,1.20]	1.06	[0.94,1.21]
$35,000–$49,999	1.12	[0.93,1.34]	1.12	[0.93,1.34]
$50,000 & Over	1.17	[0.92,1.48]	1.17	[0.93,1.49]
Past SU Problems (Ref = None)				
Yes	1.14^***^	[1.02,1.28]	1.14^***^	[1.02,1.28]
Mental Health Problem (Ref = Ever)				
No History of Problems	1.14	[0.99,1.31]	1.14	[0.99,1.30]
Misdemeanors (Ref = 1 or Less)				
2	1.03	[0.90,1.18]	1.03	[0.90,1.18]
3–4	1.09	[0.93,1.29]	1.09	[0.93,1.29]
5 or More Misdemeanors	0.99	[0.67,1.46]	0.99	[0.67,1.46]
Recidivism Risk Level (Ref = Low)				
Moderate	0.98	[0.84,1.14]	0.98	[0.84,1.14]
Moderate-High	1.03	[0.89,1.20]	1.03	[0.88,1.19]
High	0.73	[0.60,0.89]	0.73	[0.60,0.89]
Age at 1st offense (Ref = 12 and Under)				
13 to 14	1.23^***^	[1.05,1.43]	1.23	[1.05,1.43]
15	1.38^***^	[1.16,1.65]	1.39^***^	[1.16,1.66]
16	1.24	[1.01,1.52]	1.24	[1.01,1.53]
Over 16	1.20	[0.93,1.56]	1.20	[0.93,1.55]
Observations	12,128		12,128	
Pseudo *R*^2^	0.49		0.49	

**Fig. 2:**
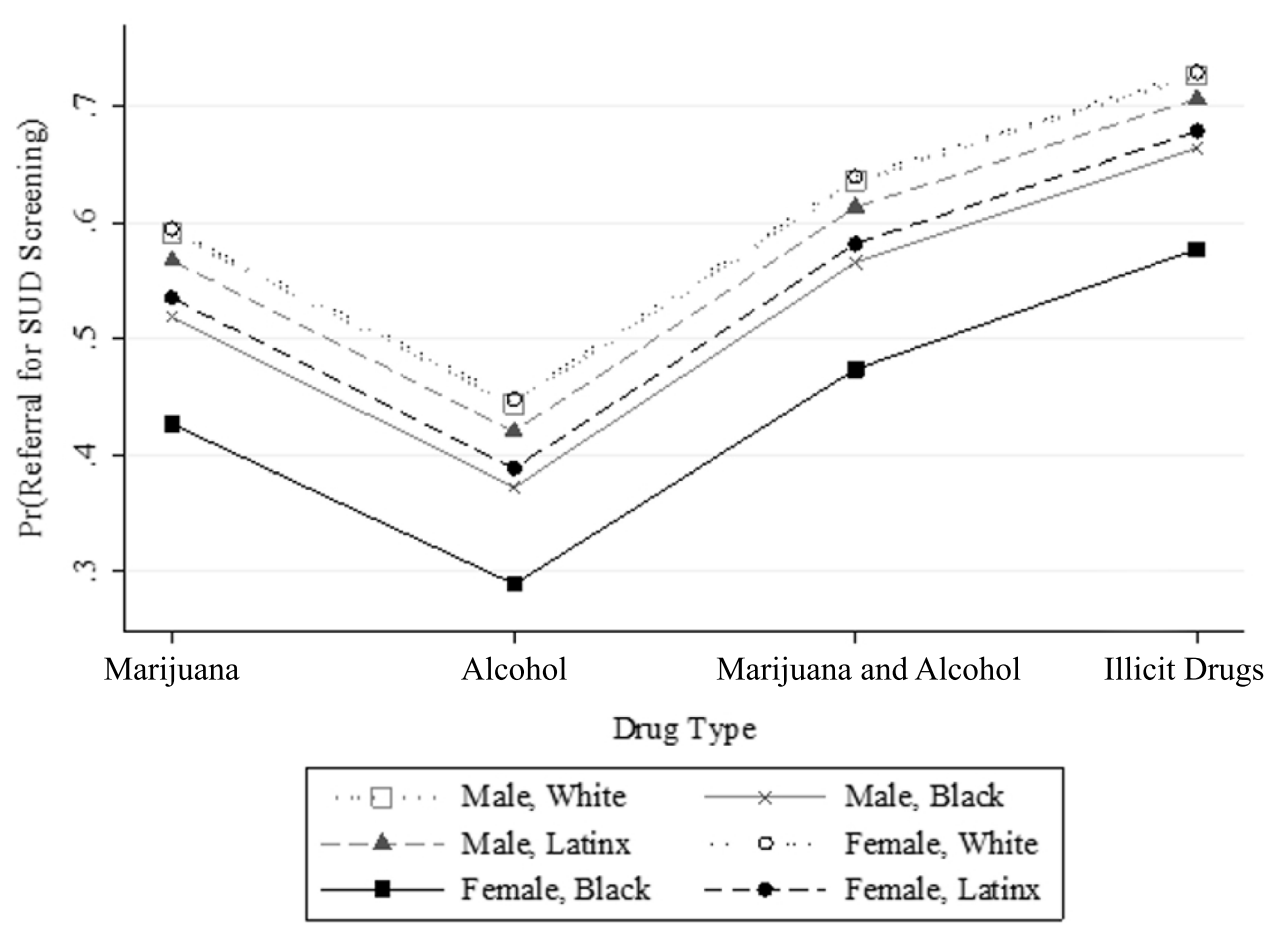
Predicted margins (Pr) of history of self-reported referrals to SUD assessment by gender, race, and drug type. *N* = 12,128 (drug type category “other drugs” not displayed)

95% confidence intervals in brackets *** *p* < 0.001. See Fig. 2 for interaction effects

Model 2 of Table 2 displays the association between the six-item gender-by-race variable and the history of self-reported referral to SUD assessment. There was no difference in the likelihood of having a history of being referred between White females and White males and no difference in the likelihood of having a history of self-reporting a referral between Latinx females and White males. However, compared to White males, Black males were 35% less likely to have a history of reporting being referred (adjusted OR: 0.65; 95% CI: 0.56–0.75), Black females were 38% less likely to have reported a referral (adjusted OR: 0.62; 95% CI: 0.49–0.79), and Latinx males were 19% less likely to report being referred (adjusted OR: 0.81; 95% CI: 0.59–1.13). In Fig. 2, the predictive margins are plotted to graphically display the interaction effects of race and gender by substance type. Fig. 2 illustrates that Black youth are less likely to have ever reported being referred than other gender-by-race groups across different types of SU profiles, but Black females suffer the greatest disparity. The drug type category “other drugs” is not displayed in Fig. 2 because specific drugs cannot be individually identified.

## Discussion

This study aimed to investigate the potential interaction effects of race and gender on the likelihood of self-reporting a referral to SUD assessment among JIC who report using substances. Despite national efforts to ensure that adolescents who report current SU are referred for SUD assessment, there were noteworthy racial and gender disparities in youth reported referrals to SUD assessment. Black females who reported current SU were 35% as likely to have -reported being referred to SUD assessment as White males and females who reported current SU while controlling for substance type, household income, age, current SU problems, history of mental health problem, total misdemeanors, and recidivism risk level.

Disparities in SUD assessment and services among minoritized girls can have devastating consequences, especially during the current drug epidemic in the U.S. Black women are foundational in the Black community and play a vital role in the social fabric of the US and around the world [45–47]. If the wellness of Black girls is diminished, it can devastate the Black community and proliferate to affect populations across the US adversely. The disparity in self-reported referral to SUD assessments may indicate a lack of access to services for minoritized populations. This could be due to racial and gender biases that favor males and Whites, such that Black girls are at the lowest tier of the preference hierarchy [48]. These disparities could also be due to a presumption of resilience and low risk among Black girls, especially when compared to White boys and White girls. The presumption of resilience among Black girls is reflective of the “Strong Black Woman” stereotype or “Black Superwoman Scheme” that portrays Black women as strong and resilient to life stressors and trauma [49–51]. However, research suggests that these phenomena play a detrimental role in harming Black women’s emotional, mental, and physical health [49].

Immediate steps can be taken to reduce disparities in self-reported referrals for SUD assessment. The protocols and mandates in the justice system must also extend to other arenas that serve youth. Based on the OJJDP reform policy, institutions serving adolescents can reduce disparities by referring all children for SUD assessment when SU is reported and evaluating disparity reduction progress via independent researchers. The Hal S. Marchman Alcohol and Other Drug Services Act of 1993, or the Marchman Act, provides voluntary or involuntary emergency assistance and temporary detention for individuals requiring substance use evaluation and treatment [52]. The Marchman Act specifically prioritizes JIC who are substance misuse offenders, substance misuse impaired at the time of arrest, second or subsequent offenders, or minors who are taken into custody [52]. Some juvenile justice institutions have made extraordinary progress toward reducing disparities and addressing the mental health needs of the children they serve with initiatives such as Civil Citations [53], the Juvenile Detention Alternatives Initiative (JDAI) [54], the Juvenile Justice System Improvement Project (JJSIP) [55], and Juvenile Justice – Translational Research on Interventions for Adolescents in the Legal System (JJ-TRIALS). JJ-TRIALS is a data-driven initiative that incorporates elements of the Juvenile Justice Behavioral Health Services Cascade framework [34, 35]. Justice communities across the nation must invest in systematic data collection and analysis by extramural researchers to monitor and evaluate the effectiveness of their safeguards.

Given the precautions, redundancies, and checks within the juvenile justice systems, senior officials and experts in the juvenile justice system have pointed out that it is virtually impossible that these disparities are occurring within the juvenile justice system, but rather are likely occurring in the youth’s community. Schools, medical facilities, and other institutions may help to reduce disparities by establishing similar mandates, data-driven disparity reduction protocols, and collaborations with independent researchers to assess progress [56]. Researchers and community organizations must work together to identify the specific factors producing these disparities. These disparities may be due to a lack of culturally relevant or gender-responsive programming. Interpersonal biases may play a major role, and stakeholders must expand access to resources that reduce biases.

## Limitations

The results of this study provided crucial insights, illustrating disparities in youth-reported referrals for SUD services in the critical moment of a national substance misuse epidemic. This study was the first to leverage statewide data to investigate the interaction effects of race and gender on the likelihood of ever self-reporting a referral for SUD assessment among JIC in Florida – the third largest state in the US. The sample was large and racially/ethnically diverse, with an adequate population of females. Despite its scientific merit, the study had some limitations.

The primary outcome variable was a self-reported measure of a youth’s history of being referred to service. It does not specify a particular system or institution that failed to make a referral. Because the FLDJJ has a referral process with two levels of quality control (supervisor sign-off and Monitoring and Quality Improvement checks), it is highly unlikely that observed disparities in self-reported substance abuse referrals occurred in the juvenile justice system. However, this sample of youth offers some insight into the experiences of delinquent and at-risk youth in the community. Future research should leverage clinical or official records to measure referral to services. The dataset also had limited information on SU and SUD. Therefore, several cases were categorized as other drug types, and limited responses on the types of drugs used warranted collapsing several drugs into a single category, illicit drugs. Future studies should include comprehensive and detailed information, including data on initiation, diagnoses, referral source, and frequency. The PACT assessment is administered by trained FLDJJ staff. Therefore, some JIC may underreport potentially incriminating information. This may have resulted in JIC underreporting SU and explain why 67% of JIC in this sample did not report SU and why certain groups are less likely to report referrals. To improve respondent veracity among correctional populations, intake assessments should be administered by social workers or mental health counselors whom youth feel comfortable with. Certain minoritized groups were underrepresented in the data, some of which may suffer harsher consequences from racial disparities in SUD services. FLDJJ uses the Massachusetts Youth Screening Instrument – Second Version (MAYSI-2). Some scholars suggest incorporating other validated screening tools during intake, such as CRAFFT [57] or UNCOPE [58, 59], to further assess substance abuse and risk for SUD to address response bias. For example, future research should investigate disparities in services among Native American populations. Despite these limitations, this study found substantial evidence that certain groups report being referred less than others. The study also provided practical recommendations to reduce SU and health disparities among adolescents.

## Conclusion

However, the findings of this current study show the elusiveness, complexity, and resilience of racial and gender disparities. Even in the context of state and federal efforts to reduce disparities, we still observe apparent differences in self-reported referrals. Among a sample of JIC who reported substance use, Black females were less likely to self-report ever being referred to SUD assessment than White males and White females. According to FLDJJ, there are strong processes in place, and experts maintain that these disparities likely occurred prior to the system or are due to alternative explanations. This disparity in self-reported referral to SUD assessment reduces access to potentially life-saving treatment services for Black females, which can lead to SUD, overdose, and related adverse health outcomes. These necessitate a further investigation of potential disparities in community samples and the implementation of disparity-reduction protocols in schools, medical facilities, and other institutions that serve youth.

## Data Availability

The research reported in this publication was approved by the Institutional Review Boards at the University of South Florida (STUDY001497) and the Florida Department of Juvenile Justice (FLDJJ). The data are not publicly available. Request for access must be made directly to FLDJJ. Several articles have been published using these data. However, the authors are not aware of any research which has used these data to investigate the specific relationships that are examined in the present study.

## Abbreviations

FLDJJ: Florida Department of Juvenile Justice
JIC: Justice-involved children
SU: Substance use
SUD: Substance use disorder
US: United States
